# To What Extent Do Free Healthcare Policies and Performance-Based Financing Reduce Out-of-Pocket Expenditures for Outpatient services? Evidence From a Quasi-experimental Study in Burkina Faso

**DOI:** 10.34172/ijhpm.2022.6767

**Published:** 2022-12-28

**Authors:** Thit Thit Aye, Hoa Thi Nguyen, Stephan Brenner, Paul Jacob Robyn, Ludovic Deo Gracias Tapsoba, Julia Lohmann, Manuela De Allegri

**Affiliations:** ^1^Heidelberg Institute of Global Health, Medical Faculty, University of Heidelberg, Heidelberg, Germany.; ^2^Health, Nutrition and Population Global Practice, World Bank, Washington, DC, USA.; ^3^National Institute of Public Health, Ouagadougou, Burkina Faso.; ^4^Department of Global Health and Development, London School of Hygiene & Tropical Medicine, London, UK.

**Keywords:** Health Financing, Out-of-Pocket Expenditures, User Fee Removal, Performance-Based Financing, Burkina Faso, Universal Health Coverage

## Abstract

**Background:** Burkina Faso has been implementing financing reforms towards universal health coverage (UHC) since 2006. Recently, the country introduced a performance-based financing (PBF) program as well as user fee removal (*gratuité*) policy for health services aimed at pregnant and lactating women and children under 5. We aim to assess the effect of *gratuité* and PBF policies on facility-based out-of-pocket expenditures (OOPEs) for outpatient services.

**Methods:** Our study is a controlled pre- and post-test design using healthcare facility data from the PBF program’s impact evaluation collected in 2014 and 2017. We compared OOPE related to primary healthcare use incurred by children under 5 and individuals above 5 to assess the effect of the *gratuité* policy on OOPE. We further compared OOPE incurred by individuals residing in PBF districts and non-PBF districts to estimate the effect of the PBF on OOPE. Effects were estimated using difference-in-differences models, distinguishing the estimation of the probability of incurring OOPE from the estimation of the magnitude of OOPE using a generalized linear model (GLM).

**Results:** The proportion of children under 5 incurring OOPE declined significantly from 90% in 2014 to 3% in 2017. Concurrently, mean OOPE also decreased. Differences in both the probability of incurring OOPE and mean OOPE between PBF and non-PBF facilities were small. Our difference in differences estimates indicated that gratuité produced an 84% (CI -86%, -81%) reduction in the probability of incurring OOPE and reduced total OOPE by 54% (CI 63%, 42%). We detected no significant effects of PBF, either in reducing the probability of incurring OOPE or in its magnitude.

**Conclusion:** User fee removal is an effective demand-side intervention for enhancing financial accessibility. As a supply-side intervention, PBF appears to have limited effects on reducing financial burden.

## Background

**Key Messages**

** Implications for policy makers**

- In Burkina Faso, user fee removal proved to be effective in reducing the probability of incurring any positive out-of-pocket expenditure (OOPE) as well as the magnitude of OOPE.
- We found no evidence of performance-based financing (PBF) reducing either the probability of incurring OOPE or the magnitude of OOPE.
- PBF appeared to have no additional benefit to the user fee removal policy in terms of financial protection.

** Implications for the public**

User fees are a major barrier to seeking healthcare in low- and middle-income countries. Our study shows that the national user fee removal policy in Burkina Faso was effective in reducing both the probability of incurring out-of-pocket expenditure (OOPE) and the amount of OOPE. Evidence from other settings has indicated that this effectiveness cannot be taken for granted, as substantial OOPE persist in other similar contexts. More studies are needed to understand key success factors for the implementation as well as to explore whether there are any transportation and drug expenses outside the healthcare setting, that impose a financial burden on vulnerable populations. In contrast, we did not find any evidence of performance-based financing (PBF) reducing OOPE. Therefore, increasing healthcare provider revenues alone may not be sufficient to influence how users are charged at the point of care. Hence, PBF may not be an effective means of promoting financial protection for vulnerable populations.

The ambition to reach universal health coverage (UHC) sits at the core of Sustainable Development Goal 3, largely defining our global strategy to improve health and well-being for all by 2030. In turn, health system strengthening, including strengthening of health financing structures, is fundamental to achieving UHC.^1^ Different indicators are used to monitor progress towards UHC, capturing inclusion in social health protection mechanisms, access to quality health services, and financial protection, measured in relation to reduced out-of-pocket spending as incurred during the process of seeking care.^2^

 User fees, ie, direct payments for care at point of use, represent an important barrier to access needed healthcare services in case of illness,^3^ and ultimately hindering countries from moving closer to UHC. For instance, women in Mali experienced delays in seeking antenatal care^4^ and treating their children^5^ because of user fees. Besides, user fees have also been observed to increase the risk of facing a catastrophic expenditure in the process of seeking care. A study in Uganda found that nearly one-third of households faced financial catastrophe after surgery.^6,7^ The financial risks derived from user fees are higher among disadvantaged groups such as the poor, people from low-income groups, or in rural areas.^7-9^

 Burkina Faso is among the many countries in West Africa to have implemented multiple health financing reforms towards UHC. The country policy journey started with the introduction of a user fee reduction policy for obstetric care in 2006,^10^ reducing user fee payments from 100% to the equivalent of 20% of the total cost of delivery. Between 2006 and 2016, a number of international organizations piloted complete user fee removal for women and children in different areas of the country.^11^ Finally in June 2016, the *gratuité* policy was launched nation-wide, calling for the removal of user fees for pregnant and lactating women and children under 5.^11^ Alongside efforts aimed at lifting financial barriers, the country also invested in a performance-based financing (PBF) program, engaging health providers in performance contracts to increase both the quantity and the quality of service delivery. After a pre-pilot project launched in 2011, PBF was piloted in twelve districts distributed over 6 regions from 2014 to 2018. The country’s commitment towards UHC is elucidated by current efforts towards establishing a national health insurance scheme, the *Caisse Nationale d’Assurance Maladie Universelle* (CNAMU), which is expected to integrate strategic purchasing arrangements, based on the experience of the prior PBF program.^12,13^

 The abovementioned efforts aimed at strengthening health financing towards UHC in Burkina Faso have been accompanied by substantial research. A number of studies have been conducted to assess the impact of user fee removal pilots,^3,14-25^ PBF,^26-34^ and more recently the *gratuité.*^31,35-40^ In general, however, existing studies have focused primarily on assessing the effects of policy reforms on equitable access to quality healthcare rather than on financial protection.^18-20,22,27,35-37^ Very few studies assessed the financial protection granted by the *gratuité*policy^35-37^ and by PBF.^27^ Under the *gratuité* policy, studies found that a third of women paid out-of-pocket expenditure (OOPE) while seeking maternal healthcare^35^ and 10% of respondents paid OOPE to acquire drugs and supplies that were supposed to be free.^37^ Following the discontinuation of user fee exemption combined PBF program in 2018, ultra-poor households experienced OOPE when receiving free healthcare at public facilities.^27^ Moreover, what we witness in the context of Burkina as elsewhere in the world, is that health financing reforms implemented in parallel, possibly to address simultaneously demand- and supply-side determinants of access to care, are often evaluated separately as if they experienced no interaction in the everyday practice of health service delivery.^41^ Again, this may be due to structural limitations in research practice, but it is nonetheless worrisome since it may lead to erroneous policy recommendations.

 In light of the abovementioned evidence gaps and of the specific policy context of Burkina Faso, our study addressed 3 related research questions, targeting the financial protection dimension of both the *gratuité* and the PBF policy. More specifically, our study aimed to assess (1) the effect of *gratuité*policy on OOPE, (2) the effect of PBF intervention on OOPE, and (3) the combined effect of *the gratuité* and PBF on OOPE for outpatient services. Moreover, our study aims to generate baseline values against which to assess further financial protection gains generated by CNAMU.

###  Conceptual Framework

 Our analysis is rooted in our understanding of the two policies and their expected effects, reflecting our working hypotheses, as described hereafter.

 Low- and middle-income countries have been increasingly implementing user fee removal policies to remove financial barriers at point of care to improve health service utilization while also enhancing financial protection.^42^ Although existing evidence suggests that in most contexts user fee removal policies do not necessarily fully alleviate the financial burden on households,^22,43-47^ the literature on Burkina Faso indicates that in this context targeted user fee exemption policies and pilot interventions have been comparatively effective in reducing OOPE.^18,19,35^ In light of existing evidence emerging from the previous policies and interventions, we postulated that by lifting payments for consultation, medicines, and laboratory examinations at point of use, the nation-wide *gratuité*policy should also have resulted in reduced OOPE for the targeted groups, children under 5 in our specific case. Nonetheless, we did not expect OOPE to drop to zero, given the concern that providers may still charge for some selected procedures. Likewise, we did not expect OOPE to decline for non-targeted groups, such as adults seeking curative consultations.

 PBF is a supply-side-focused intervention aimed at improving the quantity and quality of health services. As described earlier, in Burkina Faso, PBF payments were added to traditional input-based financing in contracted facilities to increase overall revenues for health facilities.^28^ Given that the goal of PBF is not to reduce financial barriers, and only a very small percentage of the population was identified as ultra-poor and as such targeted by specific equity measures, we did not expect the latter to produce a substantial decline in OOPE among individuals seeking care at PBF facilities. Nonetheless, we did expect PBF to result in a moderate decline in OOPE. In Burkina Faso, the management committees of the health facilities, ie, known as COGES (les comités de gestion), are granted autonomy in defining consultation fees and in lifting payments for the ultra-poor.^48,49^ Therefore, we postulated that, given the additional revenues generated by PBF, health providers would have been inclined to reduce user charges for selected outpatient services and items, resulting in an overall moderate reduction in OOPE. We further postulated that, again thanks to the additional revenues and the overall management support offered by the program, PBF providers should have been in a better position to implement the *gratuité*policy,and that therefore OOPE reduction for targeted groups would be more pronounced in PBF facilities.

## Methods

###  Study Setting

 Burkina Faso is a landlocked Western African country with 20.3 million inhabitants^50^ and a gross domestic product (GDP) per capita of US$ 786.9 in 2019, in which four out of ten people still live below the national poverty line.^51^ In 2018, current health expenditure (CHE) was equivalent to 5.6% of its GDP, with contributions by the government, international donors, and OOPE being equivalent to 42.5%, 15.2%, and 35.8%, respectively.^51^ The high proportion of CHE recovered via OOPE is a reflection of the fact that the country’s health financing structure still largely relies on user fees, applied to a wide range of services.^36,52^ The literature has consistently reported that user fees continue to deter people, especially the poor, from using healthcare services.^8,19^

 Primary healthcare provision is structured along a two-tier system whereby “Centre de Santé et de Promotion Sociale” (CSPS) and medical centers (CM) represent the first points of care in rural areas, and district hospitals located in the district capital act as the second point of care.^53^

 As indicated in the introduction, the country has recently made substantial efforts to reform its health financing structures to promote greater financial access to primary healthcare for its population. With specific reference to the reforms addressed by our study, we note the PBF program and the *gratuité*policy. The PBF program was launched in 2014 after the baseline data collection and discontinued in 2018 in twelve districts distributed across 6 regions, namely Centre Nord, Centre Ouest, Nord, Nord, Sud Ouest, Boucle du Mouhoun, and Centre Est (Figure).^54^ Details of the intervention and its impact evaluation design have been described elsewhere.^13,55^ As part of the impact evaluation, 4 different PBF models were implemented in parallel whereby traditional supply-side PBF was either implemented on its own or in combination with one of 3 equity measures (ie, user fee removal targeting the ultra-poor; or user fee removal with additional provider payments for each treated ultra-poor patient; or community-based health insurance enrollment in parallel to PBF roll-out with full subsidization of insurance premiums for the ultra-poor). The PBF intervention was allocated at the district level and all CSPS and CM within the district and their catchment areas were randomly allocated to one of the 4 PBF intervention modalities.

**Figure:**
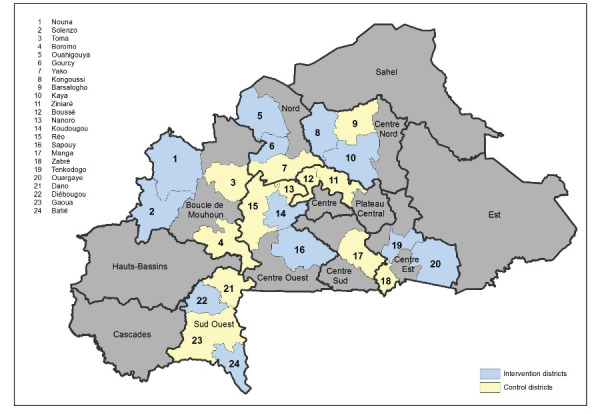
Study Districts.

 The *gratuité*policy has been implemented nationwide since June 2016. This means that across the country, women and children under 5 are entitled to receive all defined services free of charge, with the government reimbursing facilities directly for the portion of the costs normally recovered via user fees.^11,56^ For other health services, people continue to face user charges at point of use when seeking care. At the time of the study, no strategic purchasing arrangements were in place for the *gratuité* program,^13^ so facilities were reimbursed according to a fee-for-service basis for all services rendered to pregnant and lactating women and children.^37^ Our analysis on the effect of the *gratuité*on OOPE is limited to children under 5, given that our dataset did not include any information on OOPE for maternity services.

###  Design

 Our study adopted a pre- and post-test design with comparison groups, using data from the PBF impact evaluation to assess both the effect of the *gratuité*policy and that of the PBF program on financial access to healthcare. We used data collected at the health facility level in the twelve PBF intervention districts and in the twelve non-PBF districts which reflect status quo service provision. Data were collected at two time points, hereafter defined as baseline (2014) and endline (2017). To assess the effect of the *gratuité* policy, we compared OOPE among children under 5 (exposed) and individuals above 5 years of age (non-exposed) at baseline and at endline. To assess the effect of the PBF program, we compared OOPE among individuals above 5 years of age residing in the PBF intervention districts (exposed) and those residing in the non-PBF districts (non-exposed). To consider the parallel roll-out of the *gratuité* policy and its potential effect on OOPE for children under 5, we compared OOPE among children under 5 residing in the PBF intervention districts (exposed) and those residing in the non-PBF districts (non-exposed).

###  Data Sources and Samples 

 We used data from both the baseline and the endline health facility survey conducted within the framework of the PBF impact evaluation, limiting our analysis to data collected at the CSPS and CM level. Baseline data were collected from October 2013 to March 2014 before the first verification and payment of the PBF program and endline data were collected from April to June 2017.

 At both time points, a panel of 508 facilities were surveyed, of which 391 were in PBF districts (census sample) and 117 were in non-PBF districts (random sample in 1:3 ratio). At each facility, exit interviews were conducted with up to 5 children under 5 and up to 5 individuals above 5 years of age who were seeking outpatient curative services on the day of our visit at the facility. This resulted in a total sample of 4449 exit interviews among children under 5 years of age (of which, 1934 at baseline and 2515 at endline) and 4473 exit interviews among individuals above 5 years of age (of which, 2013 at baseline and 2460 at endline). Exit interviews provided data on whether a person had incurred any OOPE for consultations, medical investigations, and medicines, the actual amount disbursed during the visit, as well as data on the patient’s socio-demographic and economic characteristics (patient’s age, gender, literacy status, and social economic status) and on the characteristics of the treating health worker (cadre). We matched exit interview data with data on facility characteristics (located district, level of health facilities, and PBF status) extracted from the facility infrastructural assessment.

 As for the first outcome variable (any OOPE incurred), there are no missing data. For the second outcome (the total OOPE), there are 10% of missing data. In order to impute missing data, we cross-checked both OOPE variables to determine if any missing data had incurred OOPE. If so, we imputed with the sum of subcategories of OOPE for the same observation (ie, OOPE on consultation, laboratory, imaging, medicine, and additional fees). We had no missing data in exposure variables. For co-variates, data were missing for less than 2% of observations per variable; missing data points were imputed using means or modes for other respondents at the same data collection time point, district, intervention arm, and with the same core demographic characteristics.

###  Variables Definition and Measurement

Table 1 summarizes all variables included in the study.

**Table 1:** Definition of Variables

**Variables**	**Definition**	**Measurement**
**Outcome**		
Any OOPE	Any positive health expenditures paid during the visit	0 = did not pay OOPE, 1 = paid OOPE
Total OOPE excluding zero spending	Health expenditures during the visit including consultation fee, costs for laboratory tests and radiology, drug costs and other medical costs	Continuous variable, unit of measure in the local currency (FCFA)
**Exposure**		
Entitled to *gratuité*	Outpatients who are entitled to *gratuité*	0 = individuals above 5 years of age who are not entitled to *gratuité*, 1 = children under 5 years of age who are entitled to *gratuité*
PBF	Outpatients visited PBF intervention in primary healthcare facilities	0 = Patients who visited non-PBF facilities, 1 = Patients who visited PBF facilities
**Co-variates**		
Patient's age	Age of children under 5 and individuals above 5 years of age	Continuous variable; measured in years
Patient's gender	Gender of children under 5 and individuals above 5 years of age	0 = female, 1 = male
Literacy	Literacy status of the patient or the caregiver	0 = Illiterate, 1 = Literate
Patient's socio-economic status	Classification of patients by wealth related to the sample	1 = 1st quintile (poorest), 2 = 2nd quintile (poorer), 3 = 3rd quintile (medium), 4 = 4th quintile (less poor), 5 = 5th quintile (least poor)
Health worker cadre	Classification of health workers attending patient by their professional degree	1 = medical doctors and nurse (IDE), 2 = nurse (IB, AS), 3 = Midwife (Sage Femme/ Maieuticien d'Etat), 4 = Assistant midwife (AA, AB), 5 = Itinerant health worker (AIS)

Abbreviations: OOPE, out-of-pocket expenditure; FCFA, Central African franc; PBF, performance-based financing; IDE, Infirmier Diplômé d’Etat; IB, Infirmier Breveté; AS, Attaché de santé; AA, Accoucheuse Auxilliaire; AB, Accoucheuse Brevetée; AIS, Agent itinérant de santé.

###  Dependent Variables

 In line with our study aim, for each analysis, we examined two outcome variables. The first outcome was binary and differentiated individuals depending on whether they had incurred any OOPE in the process of seeking care at the CSPS and CM. The second outcome was conditional on having incurred a positive expenditure (hence, it excluded zeros), continuous and accounted for consultation fees, costs for laboratory tests and imaging tests, medicines, and additional expenses. Transport costs were excluded because they were not explicitly targeted by either the *gratuité*or the PBF program. OOPE is measured in the local currency, CFA francs (FCFA) (US$ 1 = 541 FCFA^57^). We purposely distinguished the two outcomes, because we wanted to distinguish the effect of the policy in fully removing OOPE from the effect of the policy on the amount of OOPE.

###  Exposure Variables and Covariates

 To fulfil the aim of examining the effect of the *gratuité*policy and of the PBF intervention, we defined two binary exposure variables. The first exposure variable differentiated individuals according to their entitlement status to the *gratuité*policy, hence classifying children under the age of 5 seeking curative services as “*gratuité*-eligible” and individuals above the age of 5 seeking curative services as “*gratuité -*non-eligible.” The second exposure variable differentiated individuals according to whether they sought care in a PBF facility, or in a non-PBF facility. In addition to the exposure variables, we also identified 5 covariates, included to improve the precision of the estimates, as described in Table 1. We used the patient’s age, sex, literacy status, socio-economic status (in 5 quintiles), and cadre of the treating health workers to control for effects of individual differences.

###  Analytical Approach

 First, we used summary statistics to describe the sample characteristics and OOPE values (ie, mean, median, standard deviation, and proportions). Then, we checked for baseline differences in exposure variables (*gratuité*and PBF) using t-test and chi-square test. Last, we employed a difference-in-differences estimation to establish the effect of the two interventions on OOPE. In line with what is described earlier, we first estimated the probability of having incurred a positive OOPE and we then estimated changes in the OOPE magnitude. The estimates from the first outcome variable were beta coefficients and were interpreted as probabilities; the estimates from the second outcome with log link function were transformed into the exponential coefficients that were interpreted as a ratio of arithmetic means. This can also be expressed as the percentage changes in total OOPE in relation to change in exposure.^58^

 Accordingly, we used an ordinary least square regression to estimate the first outcome variable, the probability of incurring a positive OOPE. The equation was formulated as followed:


$$Y=\beta 0+\beta 1.Year+\beta 2.Intervention+\beta 3.\left[Intervention*Year\right]+\beta 4.Co\mathrm{var}iates+\varepsilon$$

where *Y* represents the beta coefficient of any OOPE incurred for an individual from a health facility at a time which is then converted into probability for the interpretation; *β* are coefficients; *Year* is a dummy variable that takes 0 for baseline and 1 for endline; *Intervention* is a dummy variable for *gratuité* or PBF intervention when equal to 1; *Covariates* is a set of covariates for patient and health worker characteristics as defined earlier; *ε* is the error term.

 Health expenditures are usually positively skewed as being mentioned in the literature.^58-61^ We found that our data were also highly skewed when zero spending was included. Therefore, we excluded zero spending and used a generalized linear model (GLM) gamma regression with a log link function to obtain a more accurate estimation. The equation for GLM model took the form:


$$\ln\left[E\left(Y\left|X\right.\right)\right]=\beta 0+\beta 1.Year+\beta 2.Intervention+\beta 3.\left[Intervention*Year\right]+\beta 4.Covariates$$

where ln[*E*(*Y*|*X*)] is the log link function of total OOPE per unit. Other notations have the same definitions as the previous equation.

 In line with our abovementioned ambition to estimate both the effect of the *gratuité* and that of PBF, we estimated 3 different sets of models. The first set assessed the effect of the *gratuité* policy by comparing OOPE among children under 5 and individuals above 5 years of age at baseline and endline. In addition to the covariates, we clustered the variables at facility level. As a sensitivity analysis, we estimated this first model also on a stratified sample, differentiating individuals seeking care at PBF and at non-PBF facilities. The second set assessed the effect of PBF by comparing OOPE among individuals above 5 years of age in PBF facilities and non-PBF facilities at baseline and endline. The third set assessed the joint effect of *gratuité*and PBF by comparing OOPE among children under 5 in PBF facilities and non-PBF facilities. In line with existing literature, for the second and the third sets of models, those assessing the effects of PBF, we applied district-level clustering to reflect the assignment of PBF as treatment to the districts.^62^ Additionally, we used facility fixed effects in all models for controlling time-invariant facility characteristics. Our study used StataIC-16 for the analysis.

## Results

Table 2 summarizes the sample characteristics. The average age of the children under 5 was 1.5 years while the average age of the individuals above 5 years of age was around 25 years. The majority of caregivers for the children under 5 were illiterate both in 2014 (87.9%) as well as in 2017 (82.9%), whereas the majority of individuals above 5 years of age were literate (93.2% in 2014 and 75.4% in 2017). One-third of the sampled individuals in 2014 consulted with doctors or nurses and the figures increased to almost half in 2017.

**Table 2:** Sample Descriptive Statistics

**Sample scale**	**Children Under 5 Years of Age **		**Individuals Above 5 Years of Age**	
**Year**	**2014**	**2017**	**2014**	**2017**
Number of observations	1934	2515	2013	2460
PBF				
From non-PBF facilities	359 (18.6%)	581 (23.1%)	411 (20.4%)	565 (23.0%)
From PBF facilities	1575 (81.4%)	1934 (76.9%)	1602 (79.6%)	1895 (77.0%)
Patient's age in years				
Mean	1.5	1.6	24.4	25.7
SD	1.2	1.2	15.4	16.4
Patient's gender				
Female	853 (44.1%)	1152 (45.8%)	1141 (56.7%)	1407 (57.2%)
Male	1081 (55.9%)	1363 (54.2%)	872 (43.3%)	1053 (42.8%)
Literacy				
Illiterate	1700 (87.9%)	2086 (82.9%)	136 (6.8%)	604 (24.6%)
Literate	234 (12.1%)	429 (17.1%)	1877 (93.2%)	1856 (75.4%)
Health worker cadre				
Medical doctor/nurse (IDE)	703 (36.3%)	1206 (48%)	798 (39.6%)	1192 (48.5%)
Nurse (IB, AS)	516 (26.7%)	544 (21.6%)	512 (25.4%)	504 (20.5%)
Midwife	18 (0.9%)	65 (2.6%)	26 (1.3%)	82 (3.3%)
Assistant midwife (AA, AB)	166 (8.6%)	99 (3.9%)	187 (9.3%)	138 (5.6%)
AIS	531 (27.5%)	601 (23.9%)	490 (24.4%)	544 (22.1%)

Abbreviations: SD, standard deviation; IDE, Infirmier Diplômé d’Etat; IB, Infirmier Breveté ; AS, Attaché de santé; AA, Accoucheuse Auxilliaire; AB, Accoucheuse Brevetée; AIS, Agent itinérant de santé.

Table 3 shows that the proportion of patients reporting any OOPE declined from more than 90% in 2014 to 3% in 2017 among children under 5, while it declined only from 94.7% to 88.1% among individuals above 5 years of age. The pattern remained unchanged even when differentiating observations at PBF and non-PBF facilities. In 2014, the mean OOPE among individuals above 5 years of age was 3 times as large as the mean OOPE among children under 5 years of age (472.5 FCFA vs 1446.7 FCFA). By 2017, this difference increased even further (353.3 FCFA vs 1509.2 FCFA). Negligible differences were observed between PBF and non-PBF facilities.

**Table 3:** Out-of-Pocket Expenditures by Year, Age Group and PBF Status: Descriptive Statistics

**I. Children Under 5 Years of Age vs Individuals Above 5 Years of Age**						
	**Children Under 5 Years of Age**			**Individuals Above 5 Years of Age**		
	**2014**	**2017**	**Difference**	**2014**	**2017**	**Difference**
Number of observations	1934	2515		2013	2460	
Any OOPE, n (%)	1753 (90.6%)	75 (3.0%)	87.6%^a^	1907 (94.7%)	2167 (88.1%)	6.6%^a^
**Total OOPE excluding zero spending**						
Number of observations	1753	75		1907	2167	
Mean (in FCFA)	472.5	353.3	119.2^a^	1446.7	1509.2	-62.5
SD (in FCFA)	334.2	273.3		1383	1706	
Median (in FCFA)	450	300		1075	1100	
**II. Children Under 5 Years of Age, Sub-grouped by PBF vs Non-PBF Facilities**						
	**In PBF Facilities**			**In Non-PBF Facilities**		
	**2014**	**2017**	**Difference**	**2014**	**2017**	**Difference**
Number of observations	1575	1934		359	581	
Any OOPE, n (%)	1424 (90.4%)	58 (3.0%)	87.4%^a^	329 (91.6%)	17 (2.9%)	88.7%^a^
**Total OOPE excluding zero spending**						
Number of observations	1424	58		329	17	
Mean (in FCFA)	463.6	352.8	110.8^a^	510.7	355.1	155.6
SD (in FCFA)	316.8	269.2		398.9	295.4	
Median (in FCFA)	450	300		500	300	
**III. Individuals Above 5 Years of Age, Sub-grouped by PBF vs Non-PBF Facilities**						
	**In PBF Facilities**			**In Non-PBF facilities**		
	**2014**	**2017**	**Difference**	**2014**	**2017**	**Difference**
Number of observations	1602	1895		411	565	
Any OOPE, n (%)	1510 (94.3%)	1643 (86.7%)	7.6%^a^	397 (96.6%)	524 (92.7%)	3.9%^b^
**Total OOPE excluding zero spending**						
Number of observations	1510	1643		397	524	
Mean (in FCFA)	1492.8	1591.9	-99.1^c^	1271	1250	21
SD (in FCFA)	1401	1844		1298	1140	
Median (in FCFA)	1100	1150		950	1000	

Abbreviations: OOPE, out-of-pocket expenditures; n, number of observations which incurred OOPE; PBF, performance-based financing; SD, standard deviation; FCFA, Total OOPE excluding zero spending in local currency (Central African franc). The differences are calculated using *t* test, ^a^*P* < .01, ^b^*P* < .05, ^c^*P* < .1

Table 4 portrays the strong effect of the *gratuité*amongst children under the age of 5 experiencing an 84% (CI -86.2%, -81.6%) reduction in their probability of incurring OOPE compared to individuals above the age of 5. This effect remained unchanged when separately considering PBF and non-PBF facilities (Supplementary file 1). Total OOPE in 2017 after the *gratuité*intervention declined by 53.7% (CI 63.1%, 41.8%), compared to 2014. Table 4 also portrays how the PBF intervention led to no substantial reductions, either in the probability of incurring a positive expenditure, -2.4% (CI -8.2%, 3.4%) or in its magnitude, 3.9% (CI -21.1%, 36.9%). This finding remained consistent even when considering the simultaneous effect of PBF and *gratuité*.

**Table 4:** Difference in Differences Estimates for Out-of-Pocket Expenses in Primary Healthcare Facilities

**Any Out-of-Pocket Expenses**	**Coefficient**	**95% CI**
Estimated effect of the *gratuité*policy	-0.840^a^	-0.862, -0.816
Estimated effect of PBF	-0.024	-0.082, 0.034
Estimated effect of the *gratuité*policy and PBF interaction	0.017	-0.113, 0.147
**Total OOPE Excluding Zero Spending**	**Coefficient (Exponentiated)**	**95% CI**
Estimated effect of the *gratuité*policy	-0.769 (0.463)^a^	-0.997, -0.541 (0.369, 0.582)
Estimated effect of PBF	0.038 (1.039)	-0.237, 0.314 (0.789, 1.369)
Estimated effect of the *gratuité*policy and PBF interaction	0.764 (2.147)^b^	0.225, 1.303 (1.252, 3.681)

Abbreviations: OOPE, out-of-pocket expenditures; PBF, performance-based financing; CI, confidence interval. ^a^*P* < .01, ^b^*P* < .05. Note: See Supplementary files 2 and 3 for the full models.

## Discussion

 Our study makes an important contribution to the literature as the first study to assess the effect of both the *gratuité*policy and PBF on OOPE of children under 5 years of age in Burkina Faso. The value of its contribution lies both in its focus on examining financial protection, as few studies assessing the effect of UHC policies do, and in the choice to appraise two policies implemented in parallel at once, moving away from the exclusive focus on one policy as performed in most impact evaluations. Our findings indicate that while the *gratuité*resulted in a substantial reduction in OOPE, payments at point of care were not affected in a significant manner by PBF. This latter finding is consistent across strains of analysis, as we noted no differential effect of the *gratuité*policy in PBF and non-PBF facilities nor a reduction in OOPE among population groups not targeted by PBF.

 Looking more specifically into the effect of the *gratuité*policy, we note both a significant reduction in the proportion of children under 5 incurring any OOPE and a reduction in the magnitude of the expenditure among those reporting OOPE. The fact that the proportion of children reporting a positive OOPE declined by around 85% represents a major achievement of the *gratuité*policy. Additionally, while it may appear worrisome that some children still faced some expenditure, we wish to note that those were only a minority, about 3% of all surveyed children, and that the magnitude of the expenditure declined substantially. This percentage appears far below what has been observed in other contexts^46,47,63-67^ and probably speaks for a relatively good implementation fidelity.^68^ Nonetheless, health authorities may need to investigate the causes of remaining payments at point of care and strategies to further expand a faithful implementation of the *gratuité*policy at all public primary healthcare facilities. Furthermore, we need to note that our measure of OOPE only captures expenditures incurred at the facility level, hence we cannot exclude the possibility that total OOPE might have been higher if individuals were instructed to purchase drugs or materials outside the health facility. Further research at the population level is needed to confirm results from our study, especially in light of the fact that National Health Accounts suggest that after experiencing a decrease in 2016 and 2017, the proportion of CHE recovered through OOPE was back to pre-*gratuité* values by 2018,^69^ ie, approximately 36%.

 Overall, our findings are in line with evidence emerging from evaluations of the early user fee removal pilots implemented in the country^18-20^ as well as a recent study on the *gratuité*policy by Meda et al conducted in 2016.^35^ However, our findings are somewhat surprising considering that Meda et al revealed that one third of all women continued to experience OOPE for obstetric services, in spite of the presence of the *gratuité.*^35^ Since no other study has assessed the effect of *gratuité*on OOPE nor more simply OOPE in the context of the *gratuité*, the study by Meda et al represents our sole opportunity for direct comparison. We postulate that the difference observed between our study and the study by Meda et al may be attributable to two factors. First, while Meda et al focused on women as the target population of the *gratuité*, we focused on children under 5. One could argue that it may be easier for providers to comply with removal of all charges for relatively simple services, such as outpatient child services, rather than for more complex ones, such as obstetric care, where greater uncertainty may prevail, hence increasing the probability of facing unexpected costs.^70,71^ Second, while the study by Meda et al took place only a few months after the launch of the *gratuité*, our data were collected approximately one year after the policy launch. This means that providers might have had a longer period to conform with the new regulations. Prior research has clearly indicated that poor knowledge and understanding of the free healthcare policies in place can act as primary source for poor policy compliance.^72^ Unfortunately, our data did not include information on OOPE for obstetric services, thus a more direct comparison with the work of Meda et al is not possible.

 Looking specifically at the effect of PBF, we note, as indicated earlier, no substantial effect either on the probability of incurring any OOPE or on the magnitude of OOPE among individuals seeking care at PBF facilities. As such, our findings contradict our original hypothesis, postulating that the increased revenues generated by PBF would have made health providers more receptive to remove user charges at point of use in general and would have enabled a better implementation of the *gratuité*policies. Having looked at both effects separately, we conclude that neither one of our original expectations manifested in reality.

 Our findings are aligned with prior evidence from Diaconu et al, reviewing that PBF might have undesirable effects on consultation fees and no effect on drug expenditures^29^ and with Chinkhumba et al,^73^ who report that PBF could not produce a substantial effect on the reduction of obstetric costs at the household level in Malawi. Three factors may stand to explain our findings. First, given its primary focus on the supply-side, PBF may be unable to produce substantial changes in OOPE. It is plausible to assume that in largely underfunded systems, the additional revenues generated by PBF may not be sufficient to enable healthcare providers to lower user charges at point of use. Second, in the case of Burkina Faso specifically, disbursement of these additional revenues to healthcare providers was so delayed^30^ as to likely jeopardize any plan to reform financial management at facility level, in spite of the existing potential to act as financially autonomous agencies, in charge of setting their own user fee levels. Evidence in support of this statement also comes from a parallel study by Beaugé et al, suggesting that even among the ultra-poor specifically targeted by the embedded equity measures, OOPE remained high throughout the PBF implementation period.^27^ Third, although the PBF was implemented in conjunction with equity measures, as in the case of Burkina Faso, our study groups were not the targeted groups of the PBF with equity measures. Keeping in mind that the purposes of PBF intervention differ from those of the *gratuité*policy, saying *gratuité* works better than PBF on OOPE reduction does not seem a fair comparison.

 Similar to what was noted earlier in relation to the effect of the *gratuité*, we note the limitation that emerges from relying on data collected during exit interviews. We do not know the extent to which PBF facilities might have been better equipped and hence less likely to refer users to purchase additional items at private pharmacies, ultimately resulting in lower OOPE. As such, further population-level research is needed to confirm or dispute our findings.

###  Methodological Consideration

 We acknowledge that our study has some limitations affecting the analysis. First, our study used data originally collected for the PBF impact evaluation. As such, our analytical models relied on a quasi-experimental approach. Lack of randomization could potentially affect the internal and external validity of our study. In particular, one may question the decision to assess the effect of the *gratuité*by comparing OOPE among children under 5 (*gratuité*-eligible) and individuals above 5 years of age (*gratuité*-non-eligible). However, in the absence of any better data sources, considering also that the *gratuité* was launched nationwide in June 2016 (excluding the possibility to identify a comparable group of non-exposed children under 5 to be used as comparison group), our study provides the first and only reliable estimates on the effect of the two policies on OOPE. We performed all analyses with rigor and tested robustness of our findings by performing several sensitivity analyses. Second, as noted earlier, our study may underestimate the total amount of OOPE, since some expenditure may be incurred at a later stage, once users are instructed to purchase items outside on the private market.^35^ Nonetheless, we trust the measurement validity of our tool in capturing OOPE emerging from payment of charges at point of use, which were the primary target of our evaluation. Third, we include all PBF arms as PBF interventions and may overlook the effect of PBF with user fee removal arms. Also, we may underestimate the effect of equity measures combined with PBF arms as we may falsely assume that the ultra-poor represent a higher proportion of our study population than they actually do. Fourth, the PBF intervention was allocated at the district level, hence, we used district-level-clusters during the estimation. This could over-reject the null hypothesis for the PBF estimation. However, our estimation rarely shows positive significant effects of PBF and the chance that this affects our results is negligible. Last, one could dispute our inability to check the common trend assumption, since no nation-wide data are available to check the development of OOPE among different groups over time. Based on existing literature reporting OOPE at different points in time and across locations,^19,27,74,75^ we felt confident to assume that in the absence of the *gratuité*, OOPE would have followed the same trend for individuals below and above 5 years of age. Similarly, analysis of HMIS data confirms equal utilization rates.^31^ Last, we wish to note the inability to also assess effects on OOPE for maternal care services, which was simply due to the unavailability of relevant data.

## Conclusion

 In conclusion, our findings suggest that demand-side interventions aimed at lifting user charges at point of use may be effective in reducing payments at point of care, hence enhancing financial accessibility for poor populations. While supply-side interventions may be instrumental in achieving other health system objectives, they appear to have limited reach in reducing the financial burden on households and should therefore always be implemented in conjunction with broad scale demand-side interventions, lifting financial barriers at point of use. Given that our findings reveal that non-*gratuité*-eligible individuals continue to face high OOPE, our first most immediate recommendation would be to expand population coverage through the upcoming CNAMU as soon as possible, while our second one would be to closely monitor implementation to ensure compliance with user fee removal at facility level.

## Acknowledgments

 The authors would like to thank Badholo Hermann who served as a field coordinator of the impact evaluation in Burkina Faso. For the publication fee, authors acknowledge financial support by Deutsche Forschungsgemeinschaft within the funding program “Open Access Publikationskosten” as well as by Heidelberg University.

## Ethical issues

 This work bases on secondary data analysis and all the data used in our study received ethical clearances from the ethics committee of the medical faculty of Heidelberg University, Germany (S-272/2013) and the ethical committee of the Ministry of Health in Burkina Faso (N˚2013-7-066 and N˚2015-5-071).

 The datasets are available upon request in the World Bank’s Central Microdata Catalogue (baseline: https://microdata.worldbank.org/index.php/catalog/2761 and endline: https://microdata.worldbank.org/index.php/catalog/3504).

## Competing interests

 The data used in this study were collected as part of the impact evaluation of the PBF program in Burkina Faso, which was funded by The World Bank’s Health Results Innovation Trust Fund (HRITF) through grants to Heidelberg University, Germany, and Centre MURAZ, Burkina Faso. MDA was the principal investigator (PI) of the impact evaluation. JL and SB were fully or partly funded by the HRITF grant to Heidelberg University. LDGT is an employee of Centre MURAZ. PJR is a World Bank employee and Co-PI of the impact evaluation, but participated in writing of this paper independently of his professional engagement. The analyses presented in this manuscript were performed independent of contractual obligations in the context of the impact evaluation; all researchers involved in the production of this manuscript worked independently and without involvement or payment from the World Bank for the purpose.

## Authors’ contributions

 MDA, TTA, JL, and SB conceived the study. TTA analysed and interpreted the data with the support from JL, MDA and contributions by HTN and PJR. TTA and MDA drafted the manuscript with contributions from all other authors. All authors approved the final manuscript.

## Funding

 This study used the data from the impact evaluation of the PBF program in Burkina Faso, which was funded by the World Bank through the HRITF.

## Supplementary files

Supplementary file 1. Difference in Differences Estimates for Out-of-Pocket Expenses in PBF and Non-PBF Facilities With Gratuité Intervention.

Supplementary file 2. Difference-in-Differences Estimates for Any OOPE Incurred: Full Models.

Supplementary file 3. Difference-in-Differences Estimates for Total OOPE: Full Models.
