# Supplementary material for: To What Extent Do Free Healthcare Policies and Performance-Based Financing Reduce Out-of-Pocket Expenditures for Outpatient services? Evidence From a Quasi-experimental Study in Burkina Faso
Source: Int J Health Policy Manag. 2022 Dec 28;12:6767. doi: 10.34172/ijhpm.2022.6767 (PMC10125104; doi:10.34172/ijhpm.2022.6767)
Supplement: Supplementary file 1 — Difference in Differences Estimates for Out-of-Pocket Expenses in PBF and Non-PBF Facilities With Gratuité Intervention. [file ijhpm-12-6767-s001.pdf]

Article title: To What Extent Do Free Healthcare Policies and Performance-Based Financing Reduce Out-of-Pocket Expenditures for Outpatient services? Evidence From a Quasi-experimental Study in Burkina Faso

Journal name: International Journal of Health Policy and Management (IJHPM)

Authors' information: Thit Thit Aye[1]*, Hoa Thi Nguyen[1], Stephan Brenner[1], Paul Jacob Robyn[2], Ludovic Deo Gracias Tapsoba[3], Julia Lohmann[4,1], Manuela De Allegri[1]

[1]Heidelberg Institute of Global Health, Medical Faculty, University of Heidelberg, Heidelberg, Germany.

[2]Health, Nutrition and Population Global Practice, World Bank, Washington, DC, USA.

[3]National Institute of Public Health, Ouagadougou, Burkina Faso.

[4]Department of Global Health and Development, London School of Hygiene & Tropical Medicine, London, UK.

(Corresponding author: thitthit.aye@uni-heidelberg.de)

Supplementary file 1. Difference in Differences Estimates for Out-of-Pocket Expenses in PBF and Non-PBF Facilities With *Gratuité* Intervention

**Any out-of-pocket expenses**	**coefficient**	**95% CI**
Estimated effect of the *gratuité* policy in PBF facilities	-0.830***	(-0.856, -0.803)
Estimated effect of the *gratuité* policy in non-PBF facilities	-0.865***	(-0.914, -0.816)
**Total OOPE excluding zero spending**	**coefficient (exponentiated)**	**95% CI**
Estimated effect of the *gratuité* policy in PBF facilities	-0.722 (0.486) ***	-0.988, -0.456 (0.372, 0.634)
Estimated effect of the *gratuité* policy in non-PBF facilities	-0.978(0.376) ***	-1.345, -0.612 (0.261, 0.542)
Abbreviation: PBF = performance-based financing, CI = confidence interval; *** P <0.01, ** P <0.05, * P <0.1		
