# Supplementary material for: To What Extent Do Free Healthcare Policies and Performance-Based Financing Reduce Out-of-Pocket Expenditures for Outpatient services? Evidence From a Quasi-experimental Study in Burkina Faso
Source: Int J Health Policy Manag. 2022 Dec 28;12:6767. doi: 10.34172/ijhpm.2022.6767 (PMC10125104; doi:10.34172/ijhpm.2022.6767)
Supplement: Supplementary file 2 — Difference-in-Differences Estimates for Any OOPE Incurred: Full Models. [file ijhpm-12-6767-s002.pdf]

Article title: To What Extent Do Free Healthcare Policies and Performance-Based Financing Reduce Out-of-Pocket Expenditures for Outpatient services? Evidence From a Quasi-experimental Study in Burkina Faso

Journal name: International Journal of Health Policy and Management (IJHPM)

Authors' information: Thit Thit Aye[1]*, Hoa Thi Nguyen[1], Stephan Brenner[1], Paul Jacob Robyn[2], Ludovic Deo Gracias Tapsoba[3], Julia Lohmann[4,1], Manuela De Allegri[1]

[1]Heidelberg Institute of Global Health, Medical Faculty, University of Heidelberg, Heidelberg, Germany.

[2]Health, Nutrition and Population Global Practice, World Bank, Washington, DC, USA.

[3]National Institute of Public Health, Ouagadougou, Burkina Faso.

[4]Department of Global Health and Development, London School of Hygiene & Tropical Medicine, London, UK.

(Corresponding author: thitthit.aye@uni-heidelberg.de)

Supplementary file 2. Difference-in-Differences Estimates for Any OOPE Incurred: Full Models.

Any OOPE	**Model 1 (*gratuité*)**			**Model 2 (PBF)**			**Model 3 (*gratuité* & PBF)**		
	Coef[a]	95% CI[b]		Coef[a]	95% CI[b]		Coef[a]	95% CI[b]	
Year*Intervention	-0.84***	-0.862	-0.816	-0.024	-0.082	0.034	0.017	-0.113	0.147
Year (2017)	-0.056***	-0.075	-0.037	-0.047*	-0.098	0.005	-0.902***	-1.017	-0.788
Age	0.000	0.000	0.001	0.000	-0.001	0.001	0.011***	0.004	0.019
Sex (male)	0.028***	0.017	0.038	0.050***	0.032	0.069	0.005	-0.013	0.024
Literate	0.020**	0.005	0.036	0.007	-0.024	0.038	-0.004	-0.016	0.009
Patient's socio-economic status									
Poor	0.027***	0.010	0.044	0.037**	0.004	0.071	0.011	-0.010	0.033
Medium	0.020**	0.002	0.039	0.015	-0.012	0.041	0.016	-0.013	0.044
Less poor	0.007	-0.012	0.026	0.003	-0.024	0.031	0.003	-0.022	0.028
Least poor	0.018**	-0.002	0.037	0.025	-0.009	0.059	0.006	-0.017	0.030
Health worker cadre									

Nurse (IB[c], AS[d])	-0.006	-0.031	0.019	0.000	-0.028	0.028	-0.032	-0.084	0.020
Midwife	-0.168***	-0.255	-0.081	-0.187***	-0.323	-0.050	-0.126***	-0.199	-0.054
Assistant midwife (AA[e], AB[f])	-0.148***	-0.203	-0.093	-0.122***	-0.187	-0.058	-0.152**	-0.264	-0.039
AIS[g]	-0.018	-0.043	0.007	0.004	-0.029	0.036	-0.054**	-0.104	-0.004
Constant	0.907***	0.885	0.929	0.924***	0.889	0.960	0.919***	0.880	0.958

Abbreviation: coef[a] = coefficient, CI[b] = Confidence intervel, IB[c] = Infirmier Breveté, AS[d] = Attaché de santé, AA[e] = Accoucheuse Auxilliaire, AB[f] = Accoucheuse Brevetée, AIS[g] = Agent itinérant de santé, PBF = performance-based financing; *** P <0.01, ** P <0.05, * P <0.1
