# Supplementary material for: To What Extent Do Free Healthcare Policies and Performance-Based Financing Reduce Out-of-Pocket Expenditures for Outpatient services? Evidence From a Quasi-experimental Study in Burkina Faso
Source: Int J Health Policy Manag. 2022 Dec 28;12:6767. doi: 10.34172/ijhpm.2022.6767 (PMC10125104; doi:10.34172/ijhpm.2022.6767)
Supplement: Supplementary file 3 — Difference-in-Differences Estimates for Total OOPE: Full Models. [file ijhpm-12-6767-s003.pdf]

Article title: To What Extent Do Free Healthcare Policies and Performance-Based Financing Reduce Out-of-Pocket Expenditures for Outpatient services? Evidence From a Quasi-experimental Study in Burkina Faso

Journal name: International Journal of Health Policy and Management (IJHPM)

Authors' information: Thit Thit Aye[1]*, Hoa Thi Nguyen[1], Stephan Brenner[1], Paul Jacob Robyn[2], Ludovic Deo Gracias Tapsoba[3], Julia Lohmann[4,1], Manuela De Allegri[1]

[1]Heidelberg Institute of Global Health, Medical Faculty, University of Heidelberg, Heidelberg, Germany.

[2]Health, Nutrition and Population Global Practice, World Bank, Washington, DC, USA.

[3]National Institute of Public Health, Ouagadougou, Burkina Faso.

[4]Department of Global Health and Development, London School of Hygiene & Tropical Medicine, London, UK.

(Corresponding author: thitthit.aye@uni-heidelberg.de)

Supplementary file 3. Difference-in-Differences Estimates for Total OOPE: Full Models.

	**Model 1 (*gratuité*)**			**Model 2 (PBF)**			**Model 3 (*gratuité* & PBF)**		
	Coef[a] (Exp[b])	95% CI[c] (Exp[b])		Coef[a] (Exp[b])	95% CI[c] (Exp[b])		Coef[a] (Exp[b])	95% CI[c] (Exp[b])	
Year*Intervention	-0.769 (0.463) ***	-0.997 (0.369)	-0.541 (0.582)	0.038 (1.039)	-0.237 (0.789)	0.314 (1.369)	0.764 (2.147) ***	0.225 (1.252)	1.303 (3.681)
Year (2017)	0.295 (1.343) ***	0.233 (1.262)	0.357 (1.428)	0.013 (1.014)	-0.244 (0.783)	0.271 (1.311)	-0.783 (0.457) ***	-1.219 (0.296)	-0.348 (0.706)
Age	0.01 (1.01) ***	0.008 (1.008)	0.012 (1.012)	0.002 (1.002)	0.000 (1.000)	0.004 (1.004)	0.015 (1.015)	-0.012 (0.988)	0.043 (1.044)
Sex (male)	0.053 (1.054) **	0.004 (1.004)	0.102 (1.107)	0.111 (1.117) ***	0.039 (1.04)	0.183 (1.2)	-0.002 (0.998)	-0.077 (0.926)	0.073 (1.076)
Literate	0.548 (1.729) ***	0.482 (1.62)	0.613 (1.846)	0.076 (1.079) *	0.000 (1.000)	0.152 (1.165)	0.14 (1.15) **	0.013 (1.013)	0.266 (1.304)
Patient's socio-economic status									
Poor	-0.009 (0.991)	-0.086 (0.918)	0.069 (1.071)	0.03 (1.031)	-0.12 (0.887)	0.18 (1.198)	-0.046 (0.955)	-0.185 (0.831)	0.094 (1.099)

Medium	0.041 (1.041)	-0.038 (0.963)	0.119 (1.126)	0.058 (1.059)	-0.077 (0.926)	0.192 (1.211)	-0.016 (0.984)	-0.126 (0.881)	0.094 (1.099)
Less poor	0.000 (1.000)	-0.077 (0.926)	0.078 (1.081)	-0.007 (0.993)	-0.113 (0.893)	0.099 (1.104)	-0.061 (0.941)	-0.192 (0.825)	0.071 (1.073)
Least poor	-0.025 (0.976)	-0.111 (0.895)	0.062 (1.064)	-0.033 (0.967)	-0.172 (0.842)	0.105 (1.111)	-0.04 (0.961)	-0.202 (0.817)	0.122 (1.129)
Health worker cadre									
Nurse (IB[d], AS[e])	0.023 (1.023)	-0.078 (0.925)	0.123 (1.131)	-0.004 (0.996)	-0.114 (0.892)	0.107 (1.113)	0.037 (1.037)	-0.131 (0.877)	0.205 (1.227)
Midwife	-0.159 (0.853)	-0.454 (0.635)	0.137 (1.147)	-0.227 (0.797)	-0.59 (0.554)	0.136 (1.146)	-0.057 (0.944)	-0.733 (0.48)	0.619 (1.856)
Assistant midwife (AA[f], AB[g])	-0.096 (0.908)	-0.263 (0.769)	0.07 (1.073)	-0.065 (0.937)	-0.214 (0.807)	0.084 (1.087)	-0.113 (0.893)	-0.367 (0.693)	0.141 (1.152)
AIS[h]	0.004 (1.004)	-0.087 (0.917)	0.094 (1.098)	0.05 (1.051)	-0.03 (0.97)	0.13 (1.139)	-0.014 (0.986)	-0.223 (0.8)	0.195 (1.215)
Constant	6.323 (557.02)***	6.263 (524.58)	6.383 (591.47)	6.63 (757.36)***	6.434 (622.62)	6.826 (921.25)	6.678 (794.39)***	6.586 (724.62)	6.769 (870.87)

Abbreviation: coef[a] = coefficient, Exp[b] = exponentiated coefficient, CI[c] = Confidence intervel, IB[d] = Infirmier Breveté, AS[e] = Attaché de santé, AA[f] = Accoucheuse Auxilliaire, AB[g] = Accoucheuse Brevetée, AIS[h] = Agent itinérant de santé, PBF = performance-based financing; *** P <0.01, ** P <0.05, * P <0.1
